# Editorial: Pharmacogenomics in neglected populations

**DOI:** 10.3389/fphar.2022.1081117

**Published:** 2022-11-11

**Authors:** Mariana R. Botton, Jorge Duconge, Fernanda Rodrigues-Soares

**Affiliations:** ^1^ Transplant Immunology and Personalized Medicine Unit, Hospital de Clínicas de Porto Alegre, Porto Alegre, Brazil; ^2^ Department of Pharmaceutical Sciences, School of Pharmacy, Medical Sciences Campus, University of Puerto Rico, San Juan, Puerto Rico; ^3^ Departamento de Patologia, Genética e Evolução, Instituto de Ciências Biológicas e Naturais, Universidade Federal do Triângulo Mineiro, Uberaba, Minas Gerais, Brazil

**Keywords:** pharmacogenomics (PGx), population genetics, ancestry, pharmacogenetics, drug metabolism

In this Research Topic titled “*Pharmacogenomics in Neglected Populations*”, Asiimwe and Pirmohamed present an excellent review article about the importance of accounting for ethnic diversity in warfarin pharmacogenomics. The authors highlighted the lack of proper representation in existing pharmacogenetic studies of warfarin, emphasizing on the argument that health disparities could be exacerbated when genetic polymorphisms and algorithms are not suitable for populations with ethno-geographic particularities. Consequently, the authors examined the extent to which individual races and ethnicities are represented adequately in the existing body of pharmacogenomic evidence and postulated that the prediction power of these pharmacogenetic-guided warfarin dosing algorithms will depend on the availability of data from all ethnic groups representing the genomic diversity of the entire population.

On the other hand, in a very much-needed original research article titled “*Characterization of ADME gene variation in Colombian population by exome sequencing*”, Fonseca-Mendoza et al. provided relevant whole-exome sequencing (WES) data to illustrate the genetic variability of 35 ADME pharmacogenes among Colombians as well as their genetically-inferred admixture for the first time. Unfortunately, the vast majority of published studies have been conducted on individuals of largely European ancestry, standing out the problem of the missing diversity in human genetic studies. Indeed, there is a paucity of relevant genomic data from studies on underrepresented groups. It is noteworthy that this study revolved around the importance of partially closing that knowledge gap by characterizing the pharmacogenomic profile and the major sources of genetic variability in this neglected population. Strikingly, they found that about nine out of 10 individuals from this population have at least one actionable pharmacogenetic variant in highly polymorphic ADME-related genes (e.g., *NAT2*, *CYP2B6, CYP2D6, ABCB1*, and *DPYD*), with significant allele frequency differences detected when compared to other Latinx and the global population. In their analysis, the authors also identified multiple rare missense variants, which were 2.1 times more frequent than common variants in this population and were predicted as potentially deleterious. It is important to bear in mind that the field is shifting the attention toward low-to-moderate frequency variants, which are more likely to be population specific. Accordingly, there will no longer be a need to rely on the extrapolation of findings from one population to another. Finally, this study further evidences the utility of WES for a more comprehensive assessment of pharmacogenomics and its clinical implementation.

Leitch et al. collaborated with an original article in which they highlighted the healthcare disparities for rural and tribal populations. These populations do not have easy access to the pharmacogenetic tests and novel strategies need to be developed to improve assistance in these areas. Interviews were conducted with 45 healthcare professionals, administrative staff, and informatics professionals at three different sites serving underserved and neglected populations in Montana. Participants described the need for improvements related to infrastructure, personnel, and funding to implement pharmacogenetic tests. Participants from tribal communities emphasized the historical misuse and abuse of genetic data from peoples as a barrier for implementation. Participants recognized the importance of pharmacogenetics for improvement of treatments for patients with limited access to follow-up services in remote areas. The costs related to the test was also pointed as a barrier and as a reason that could exacerbate inequities in care. On the other hand, the telehealth service was valued by the participants as a way to deliver pharmacogenetic consults with the ability to connect providers and patients to resources and expertise relevant to driving successful pharmacogenetic implementation. The results show the importance of developing strategies to facilitate the access to pharmacogenetic tests for neglected populations in order to reduce the inequities.

Tomaz et al. evaluated the nicotine metabolite ratio (NMR) in 185 Brazilian smoking patients. The individuals were classified into slow metabolizers (NMR <0.31) and normal or fast metabolizers (NMR ≥0.31). Thirty percent (30%) of the patients were classified as slow metabolizers while 70% were considered normal/fast metabolizers. Authors also showed that there was an association between the number of cigarettes smoked per day and metabolite averages. The study described categories of metabolizers in Brazilian patients under pharmacological treatments and can contribute to the personalization of smoking cessation treatments, improving clinical outcomes.

Panda et al. analyzed 1,029 genomes from Indian individuals, part of the IndiGen initiative, and reported great differences in several variants when comparing to 1,000 Genomes samples. The authors analyzed non-synonymous variants from 545 druggable kinase genes and their protein sequences. A subset of 217 variants were used for structural analysis and critical differences were found in the strength of the binding caused by variants present in Indian populations. This article also showed how common variants present in Indian populations are related to structural protein function in molecules with pharmacogenomic interest. They suggest that the healthcare system may be “Population or Individual Specific Drug System” instead of “One Size Fits for All” by using these information, and, additionally, they suggest an ADR database to understand the risk of therapies in Indian populations.

In this context, we emphasize, with this Research Topic, that efforts are being performed in order to increase pharmacogenomic evidence about neglected populations. These studies are important to define the pharmacogenomic patterns of admixed/neglected populations, in order to increase their access to suitable precision medicine. However, we also reaffirm that more studies are needed to create a better understanding of their allele frequencies, new variants (and their relation to protein function), and clinical applications to achieve adequate personalized medicine for these populations.

